# Supplementary material for: Clinical recovery of Macaca fascicularis infected with Plasmodium knowlesi
Source: Malar J. 2021 Dec 30;20:486. doi: 10.1186/s12936-021-03925-6 (PMC8719393; doi:10.1186/s12936-021-03925-6)
Supplement: Supplementary file 1 — Additional file 1: Table S1. Macaque Cohort and Experimental Summaries. Details regarding four monkey cohorts involved in the current study are summarised; three of these were experimentally infected with P. knowlesi sporozoites, and one served as control group. The cohorts are listed in the order in which experiments using these animals were performed. These animals and their longitudinal infection designs were part of a systems biology program, with iterative cohort experimentation designed to satisfy the goals of those research programs. §The non-sequential experimental numbering (E07, E33, E34, and E35) reflects the experimental numbers assigned in the MaHPIC Laboratory Information Management System. †Telemetry data were collected in E07 (only), for temperature, blood pressure, heart rate and activity level (Brady et al., manuscript in preparation). Spx is an abbreviation for splenectomy. [file 12936_2021_3925_MOESM1_ESM.docx]

| **Supplemental Table 1: *Macaca fascicularis* Cohorts for *P. knowlesi* Infection and Control Data** | | | | |
| --- | --- | --- | --- | --- |
| **Cohort Number** (^§^Experiment Number) | **Experimental Summary** | **Monkey Codes for Assigned Animals & Acute/Chronic/Normal Sacrifice information** | **Brief Experimental Description**  (*Supplementary Figs. 1-3 describe and show graphed projected and actual data*) | ^†^**Telemetry**  **Implants** |
| **1** (E07) | Pilot Experiment: *P. knowlesi*  Acute & Chronic  Infections | Animals sacrificed during an acute infection period: 11C131, 12C36, H12C59, H12C8.  Animals sacrificed during a chronic infection period:  11C166, 12C44, 12C53. | Seven *M. fascicularis* were inoculated with *P. knowlesi* sporozoites obtained from fresh mosquito salivary gland dissections, but for unexplained reasons parasitaemias did not develop. The monkeys were subsequently infected with cryopreserved *P. knowlesi* sporozoites (the same batch used for all other experiments in this table). Blood and bone marrow samples were collected for analysis at timepoint (TP) intervals throughout the course of the infections, and euthanasia and necropsies for pathology analyses were performed sequentially at selected times representing acute or chronic infections. | Yes  *Continuous telemetry data were collected* |
| **2** (E33) | Iterative Experiment:  *P. knowlesi*  Acute & Chronic Infections | Animals sacrificed during a chronic infection period:  13C90, 14C15, 14C3, H13C110. | Four *M. fascicularis* were infected with cryopreserved *P. knowlesi* sporozoites. Blood and bone marrow samples were collected for analysis at pre-determined TP intervals. The animals were euthanised and necropsied for pathology analyses within 2-3 weeks of inoculation, once the *M. fascicularis* were controlling their infections and establishing chronic infection. | No |
| **3** (E34) | Control Experiment:  Data collection from normal uninfected animals | Animals sacrificed for normal uninfected control tissue data:  13C102,13C105,  13C129 | Three *M. fascicularis* were sacrificed to provide normal control samples for analysis in conjunction with samples from the infected macaques. | No |
| **4** (E35) | Iterative Experiment:  *P. knowlesi*  Acute & Chronic  Infection | Animals sacrificed during a chronic infection period:  13C33, 13C74, H13C101, H14C17. | Four *M. fascicularis* were infected with cryopreserved *P. knowlesi* sporozoites. Blood and bone marrow samples were collected for analysis at predetermined TP intervals throughout the experiment. The animals were euthanised and necropsied for pathology analyses between days 48-50. | No |

**-**
