# Supplementary material for: Clinical recovery of Macaca fascicularis infected with Plasmodium knowlesi
Source: Malar J. 2021 Dec 30;20:486. doi: 10.1186/s12936-021-03925-6 (PMC8719393; doi:10.1186/s12936-021-03925-6)
Supplement: Supplementary file 2 — Additional file 2: Table S2. Histopathology scores for P. knowlesi-infected kra monkeys. The kra monkey codes shaded with PINK (n = 4) represent animals sacrificed during an acute phase of the infection, and the codes shaded with GREY (n = 12) represent animals sacrificed during a chronic phase of infection. The experimental number for each coded animal is also shown: E07, E33, or E35. Nineteen tissues were examined by a veterinary pathologist and scored semi-quantitatively in the categories above. Spleen, lymph nodes, and bone marrow were not scored because the changes were deemed to be physiological reactions to infection and/or anaemia. The following tissues were examined for histopathological changes, but omitted from this table because no changes were found: aorta, adrenal gland, cerebrum, cerebellum, eye, midbrain, muscle, skin, testis, thymus, and omentum. These tissues were assigned a score of zero for modeling purposes. Semi-quantitative scores are presented as a heatmap to visually represent severity, with 0 being no changes or damage, and 4 being diffuse changes. Organs were scored in several categories, including inflammation, oedema, crypt inflammation (for the gastrointestinal organs), necrosis, haemorrhage, hyperplasia (including alveolar wall thickening/interstitial hyperplasia, for the lungs, Kupffer cell hyperplasia in the liver, and glomerular hyperplasia for the kidneys), fibrosis, vasculitis, and tubular degeneration (for kidneys). The histopathology categories shown reflect the range of pathologies noted, whether for one or multiple monkeys. [file 12936_2021_3925_MOESM2_ESM.docx]

| **Supplemental Table 2 Histopathology scores for *P. knowlesi*-infected kra monkeys** | | | | | | | | | | | | | | | |
| --- | --- | --- | --- | --- | --- | --- | --- | --- | --- | --- | --- | --- | --- | --- | --- |
|  | **Kra Monkey Codes & Experimental Numbers** | | | | | | | | | | | | | | |
| **Histopathology** | 11C131-E07 | 11C166-E07 | 12C44-E07 | 12C53-E07 | 12C136-E07 | H12C8-E07 | H12C59-E07 | 13C90-E33 | 13C33-E35 | 14C3-E33 | 14C15-E33 | H13C101-E35 | H13C110-E33 | H14C17-E35 | 13C74-E35 |
| **Colon** |  |  |  |  |  |  |  |  |  |  |  |  |  |  |  |
| *Inflammation* | 2 | 0 | 1 | 1 | 1 | 1 | 1 | 1 | 1 | 1 | 1 | 1 | 1 | 1 | 1 |
| *Oedema* | 0 | 0 | 0 | 0 | 0 | 1 | 0 | 0 | 0 | 0 | 0 | 0 | 0 | 0 | 0 |
| **Duodenum** |  |  |  |  |  |  |  |  |  |  |  |  |  |  |  |
| *Inflammation* | 2 | 0 | 2 | 1 | 1 | 1 | 2 | 2 | 2 | 2 | 2 | 2 | 2 | 2 | 2 |
| **Jejunum** |  |  |  |  |  |  |  |  |  |  |  |  |  |  |  |
| *Inflammation* | 1 | 1 | 1 | 1 | 1 | 1 | 1 | 2 | 1 | 1 | 1 | 1 | 1 | 1 | 1 |
| **Stomach** |  |  |  |  |  |  |  |  |  |  |  |  |  |  |  |
| *Inflammation* | 2 | 2 | 2 | 2 | 0 | 1 | 0 | 1 | 3 | 2 | 0 | 4 | 2 | 0 | 0 |
| *Crypt Inflammation* | 0 | 0 | 0 | 0 | 0 | 0 | 0 | 0 | 0 | 0 | 0 | 0 | 0 | 0 | 0 |
| **Kidney** |  |  |  |  |  |  |  |  |  |  |  |  |  |  |  |
| *Inflammation* | 0 | 1 | 0 | 0 | 0 | 0 | 0 | 0 | 0 | 0 | 0 | 0 | 0 | 0 | 0 |
| *Haemorrhage* | 0 | 0 | 1 | 0 | 0 | 0 | 0 | 0 | 0 | 1 | 0 | 0 | 1 | 0 | 0 |
| *Tubular Degeneration* | 0 | 0 | 0 | 0 | 0 | 0 | 2 | 0 | 0 | 0 | 0 | 0 | 2 | 1 | 0 |
| *Glomerular Hypercellularity* | 1 | 1 | 1 | 1 | 1 | 1 | 1 | 1 | 1 | 1 | 1 | 1 | 1 | 1 | 1 |
| **Liver** |  |  |  |  |  |  |  |  |  |  |  |  |  |  |  |
| *Inflammation* | 2 | 2 | 2 | 2 | 2 | 2 | 2 | 2 | 2 | 2 | 2 | 1 | 2 | 2 | 1 |
| *Kupffer Cell Hyperplasia* | 2 | 2 | 2 | 2 | 3 | 3 | 2 | 2 | 2 | 2 | 2 | 1 | 2 | 2 | 2 |
| **Lung** |  |  |  |  |  |  |  |  |  |  |  |  |  |  |  |
| *Inflammation* | 0 | 0 | 0 | 0 | 0 | 0 | 0 | 0 | 0 | 0 | 0 | 0 | 0 | 1 | 2 |
| *Haemorrhage* | 0 | 0 | 0 | 0 | 0 | 0 | 0 | 1 | 2 | 0 | 0 | 1 | 0 | 0 | 1 |
| *Hyperplasia* | 1 | 0 | 1 | 1 | 0 | 2 | 1 | 1 | 1 | 1 | 1 | 1 | 1 | 1 | 2 |
| *Fibrosis* | 2 | 1 | 2 | 1 | 0 | 2 | 2 | 2 | 2 | 2 | 2 | 3 | 2 | 3 | 2 |
| **Ventricle** |  |  |  |  |  |  |  |  |  |  |  |  |  |  |  |
| *Inflammation* | 2 | 0 | 0 | 1 | 1 | 0 | 0 | 0 | 0 | 0 | 0 | 0 | 0 | 0 | 0 |
| *Oedema* | 2 | 0 | 0 | 1 | 0 | 0 | 0 | 0 | 0 | 0 | 0 | 0 | 0 | 0 | 0 |
| *Haemorrhage* | 0 | 0 | 0 | 0 | 2 | 0 | 0 | 0 | 0 | 0 | 0 | 0 | 0 | 0 | 0 |
