# Supplementary material for: Clinical recovery of Macaca fascicularis infected with Plasmodium knowlesi
Source: Malar J. 2021 Dec 30;20:486. doi: 10.1186/s12936-021-03925-6 (PMC8719393; doi:10.1186/s12936-021-03925-6)
Supplement: Supplementary file 5 — Additional file 5: Table S5. Linear regression: pathology score vs. necropsy day. The effect of time at necropsy on pathology score was tested via a linear regression model and found not to contribute. [file 12936_2021_3925_MOESM5_ESM.docx]

**Supplemental Table 5: Linear Regression: Pathology Score *vs*. Necropsy Day**

|  | | Estimate | Std. Error | *t* value | *p*-value | Significance |  |
| --- | --- | --- | --- | --- | --- | --- | --- |
| Intercept | | 0.693 | 0.208 | 3.332 | 0.001 | ** |  |
| Necropsy Day | | 0.003 | 0.006 | 0.560 | 0.576 | NS |  |
|  | *n*= 188; *df* = 190; Adjusted *R*^2^ = -0.004; *F*-statistic = 0.314; *p* = 0.576 | | | | | | |
