# Supplementary material for: Clinical recovery of Macaca fascicularis infected with Plasmodium knowlesi
Source: Malar J. 2021 Dec 30;20:486. doi: 10.1186/s12936-021-03925-6 (PMC8719393; doi:10.1186/s12936-021-03925-6)
Supplement: Supplementary file 7 — Additional file 7: Table S7. Hierarchical Linear Regression Analysis. Multiple Linear Regression (MLR) was performed to test the relationship between score, count, and organ. For organ, adrenal gland was selected as the reference tissue. Parameter is significant at p = 0.05; *p < 0.05, **p < 0.005, ***p < 0.0005.; NS = not significant. [file 12936_2021_3925_MOESM7_ESM.docx]

| **Supplemental Table 7: Hierarchical Linear Regression Analysis** | | | | | | | | | | |
| --- | --- | --- | --- | --- | --- | --- | --- | --- | --- | --- |
|  | Model 1 | | | |  | Model 2 | | | |  |
|  | Estimate | Std. Error | t value | p-value | Significance | Estimate | Std. Error | t value | p-value | Significance |
| Intercept | 0.625 | 0.115 | 5.424 | 1.78x10^-7^ | *** | -0.013 | 0.255 | -0.051 | 0.959 | NS |
| Count | 0.030 | 0.011 | 2.817 | 0.005 | ** | -0.001 | 0.007 | 0.206 | 0.837 | NS |
| Organ |  |  |  |  |  |  |  |  |  |  |
| *Aorta* |  |  |  |  |  | 0.010 | 0.353 | 0.029 | 0.977 | NS |
| *Cerebellum* |  |  |  |  |  | 0.006 | 0.351 | 0.018 | 0.986 | NS |
| *Cerebrum* |  |  |  |  |  | 0.005 | 0.351 | 0.014 | 0.989 | NS |
| *Colon* |  |  |  |  |  | 0.998 | 0.350 | 2.851 | 0.005 | ** |
| *Duodenum* |  |  |  |  |  | 1.503 | 0.350 | 4.292 | 2.97x10^-5^ | *** |
| *Eye* |  |  |  |  |  | 0.002 | 0.350 | 0.005 | 0.996 | NS |
| *Jejunum* |  |  |  |  |  | 1.108 | 0.352 | 3.147 | 0.002 | ** |
| *Kidney* |  |  |  |  |  | 1.704 | 0.350 | 4.863 | 2.62x10^-6^ | **** |
| *Liver* |  |  |  |  |  | 3.901 | 0.350 | 11.147 | <2x10^-16^ | **** |
| *Lung* |  |  |  |  |  | 3.396 | 0.350 | 9.692 | <2x10^-16^ | **** |
| *Midbrain* |  |  |  |  |  | 0.004 | 0.350 | 0.011 | 0.992 | NS |
| *Muscle* |  |  |  |  |  | 0.012 | 0.354 | 0.032 | 0.974 | NS |
| *Omentum* |  |  |  |  |  | 0.011 | 0.355 | 0.032 | 0.974 | NS |
| *Skin* |  |  |  |  |  | 0.012 | 0.355 | 0.034 | 0.973 | NS |
| *Stomach* |  |  |  |  |  | 1.602 | 0.350 | 4.577 | 9.07x10^-6^ | **** |
| *Testis* |  |  |  |  |  | 0.012 | 0.355 | 0.034 | 0.973 | NS |
| *Thymus* |  |  |  |  |  | 0.901 | 0.350 | 2.574 | 0.011 | * |
| *Ventricle* |  |  |  |  |  | 0.001 | 0.007 | 0.206 | 0.837 | NS |
|  | N = 190; df = 188; Adjusted R^2^ = 0.035; F-statistic = 7.934;  p-value = 0.005 | | | | | N = 190; df = 170; Adjusted R^2^ = 0.678; F-statistic = 21.92;  p-value = <2.2x10^-16^ | | | | |
