# Supplementary material for: Clinical recovery of Macaca fascicularis infected with Plasmodium knowlesi
Source: Malar J. 2021 Dec 30;20:486. doi: 10.1186/s12936-021-03925-6 (PMC8719393; doi:10.1186/s12936-021-03925-6)
Supplement: Supplementary file 9 — Additional file 9: Table S9. Flow cytometry staining cocktail for measuring erythroid progenitors in rhesus and kra monkey bone marrow aspirates. [file 12936_2021_3925_MOESM9_ESM.docx]

**S9 Table. Flow Antibody Cocktail**

| **Antibody** | **Fluorophore** | **Clone** | **Company** | **Titration (µl)** |
| --- | --- | --- | --- | --- |
| CD47 | FITC | MEM-122 | ThermoFisher Scientific | 10 |
| Band-3 | PE | BRIC-6 | ARP | 1 |
| CD41a | PE-Cy7 | HIP8 | Biolegend | 1.25 |
| CD71a | APC | LOI.1 | BD | 5 |
| Hoechst 33342 | Emission at 461 nm | N/A | BD | Final Concentration of 10 µg/ml |
| HLA-A,B,C | Alexfluor700 | W6/32 | Biolegend | 1.6 |
| LiveDead | Yellow | N/A | Life Technologies | 1 |
| CD45 | APC-Cy7 | D058-1283 | BD | 0.5 |
| Annexin V | BUV395 | N/A | BD | 5 |
|  |  |  |  |  |
