# Supplementary figures and images for: Clinical recovery of Macaca fascicularis infected with Plasmodium knowlesi
Source: Malar J. 2021 Dec 30;20:486. doi: 10.1186/s12936-021-03925-6 (PMC8719393; doi:10.1186/s12936-021-03925-6)

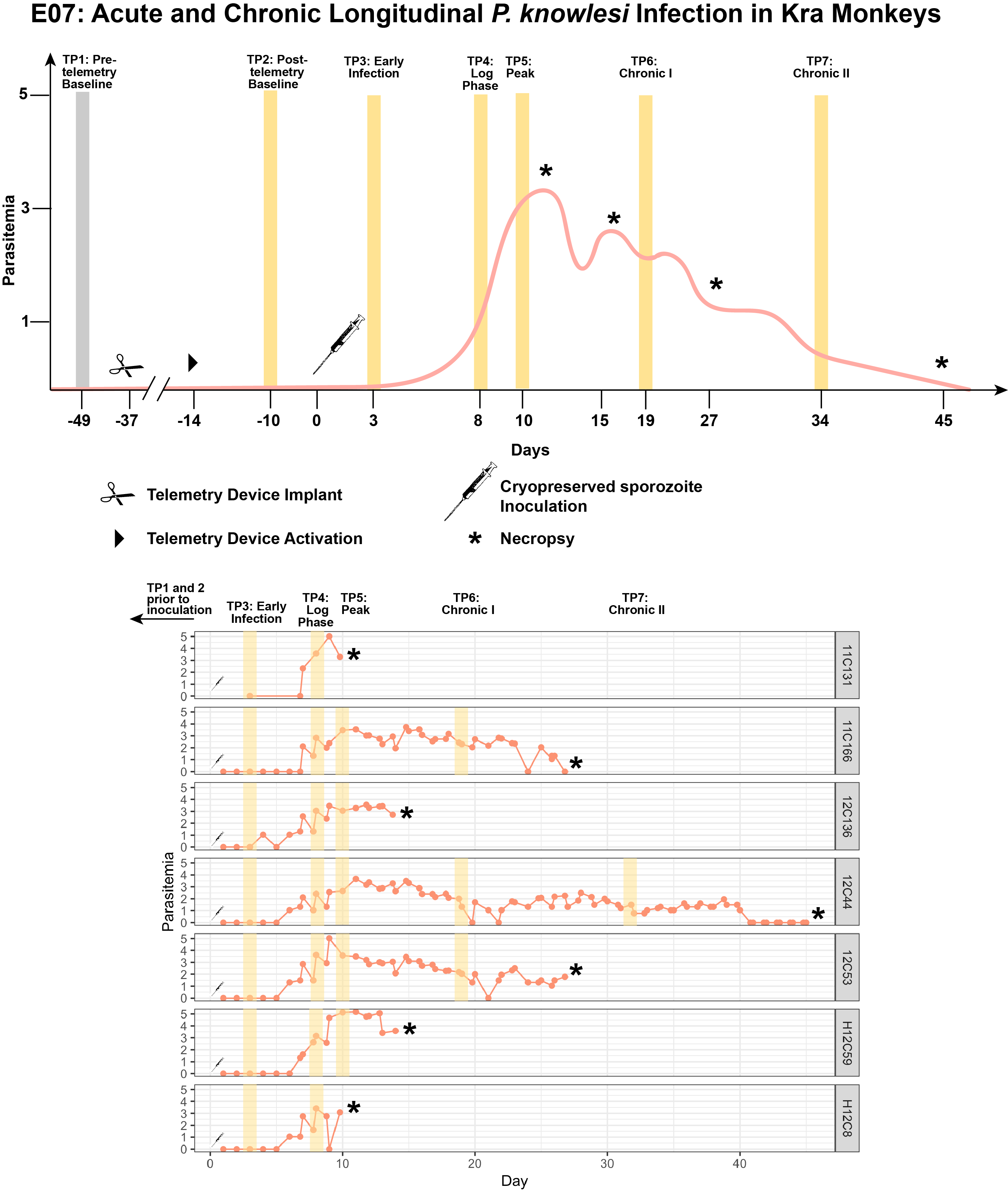

Supplement: Supplementary file 10 — Additional file 10: Fig. S1. E07 Experimental Design and Parasitaemia, as also similarly presented in PlasmoDB [91] to accompany E07 publicly available data. Pilot P. knowlesi infection in seven kra monkeys (11C131, 11C166, 12C36, 12C44, 12C53, H12C59, H12C8) with staggered necropsy endpoints that included necropsies at an acute (n = 4: 11C131, 12C36, H12C8, H12C59) or chronic (n = 3: 11C166, 12C44, 12C53) stage of infection. Top Panel: Schematic of the planned (generalized) experimental design with pre-infection surgical implantation of a telemetry device (scissors) plus recovery time and their activation for collection of physiological data, before (grey bar) and after surgery baseline timepoint (TP) sample collections (gold bars), P. knowlesi cryopreserved sporozoite inoculations at day 0 (syringe), predicted parasitaemia kinetics (pink curved line) with early infection, log-phase, peaking parasitaemia, and sequential chronic phase TPs indicated for blood and bone marrow sample collections. Necropsy endpoints (*) were planned for selected animals at an acute and early or late chronic stages of infection. Bottom Panel: Schematic showing E07 experimental data including P. knowlesi cryopreserved sporozoite inoculations on day 0 (syringe†), daily parasitaemias graphed (pink line), and defined TPs (gold bars) and the specific days of euthanasia and necropsy endpoints (*) are indicated for each of the animals. No subcurative treatments were required, as the blood-stage infections and clinical signs naturally began to resolve, as expected with kra monkeys. †This cohort had previously been inoculated with sporozoites freshly isolated from mosquito salivary glands, yet for reasons unknown, blood-stage parasitaemia did not result. Transcriptomic analysis of peripheral blood samples did not reveal any significant differences between these baseline TPs [55]. This pilot experiment allowed for (1) testing of cryopreserved stocks of P. knowlesi sporozoites and the kinetics of t [file 12936_2021_3925_MOESM10_ESM.png]

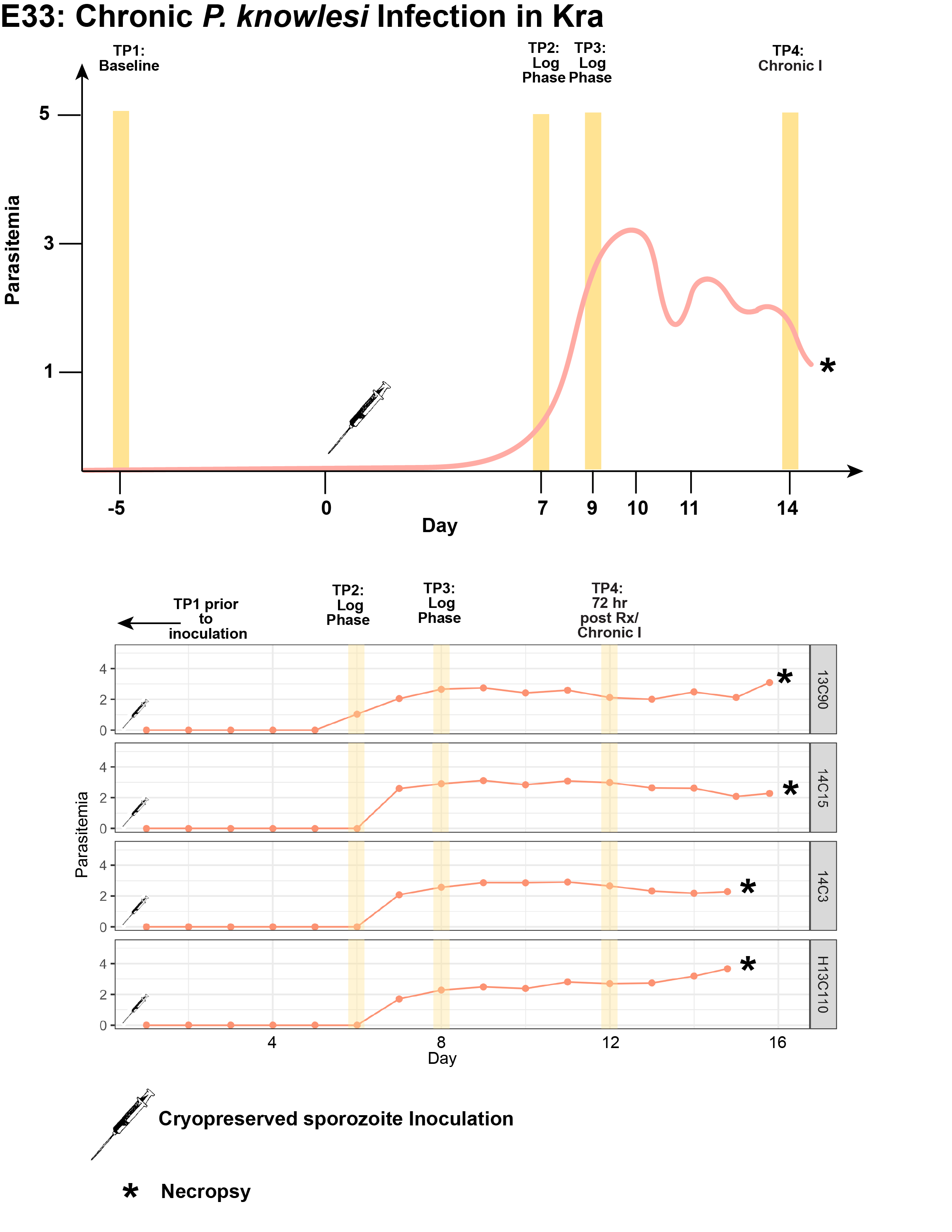

Supplement: Supplementary file 11 — Additional file 11: Fig. S2. E33 Experimental Design and Parasitaemia, as also similarly presented in PlasmoDB [91] to accompany E33 publicly available data. E33 includes iterative P. knowlesi infections in a cohort of four kra monkeys (13C90, 14C15, 14C3, H13C110) to continue to study acute and early stage “Chronic I” infections. Top Panel: Schematic of the planned (generalized) experimental design with timepoints (TP) of sample collection (gold bars), P. knowlesi cryopreserved sporozoite inoculations at day 0 (syringe), predicted parasitaemia kinetics (pink line) with early infection, log-phases, peaking parasitaemia, and later TPs indicated for blood and bone marrow sample collections. Necropsy endpoints (*) were planned for each species after day 14, early on in the natural overall decline in parasitaemia as observed for kra monkeys in E07 (Fig. S1) and again subsequently in E35 (Fig. S3). Bottom Panel: Schematic showing E33 experimental data including P. knowlesi cryopreserved sporozoite inoculations on day 0 (syringe), daily parasitaemias graphed (pink lines), and defined TPs (gold bars) and the specific days of euthanasia and necropsy endpoints (*) are indicated for each of the animals. No treatment was required. [file 12936_2021_3925_MOESM11_ESM.png]

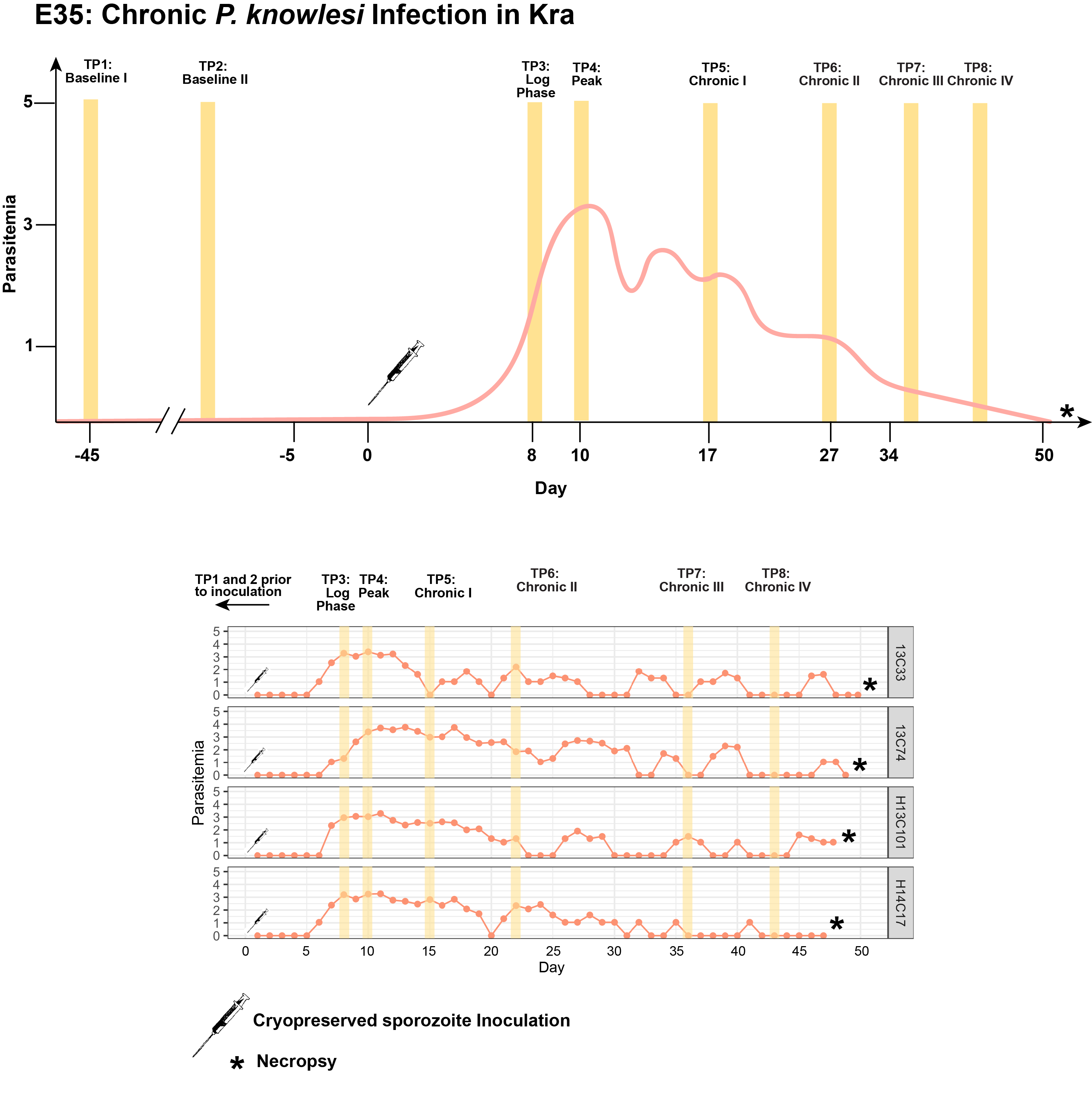

Supplement: Supplementary file 12 — Additional file 12: Fig. S3. E35 Experimental Design and Parasitaemia, as also similarly presented in PlasmoDB [91] to accompany E35 publicly available data. E35 includes iterative P. knowlesi infections in a cohort of four kra monkeys (13C33, 13C74, H13C101, H14C17) to continue to study acute and chronic infections, up to 50 days, with the later TPs 5–8 also labelled Chronic I–Chronic IV. Top Panel: Schematic of the planned (generalized) experimental design with timepoint (TP) sample collections (gold bars), P. knowlesi cryopreserved sporozoite inoculations at day 0 (syringe), predicted parasitaemia kinetics (pink line) with early infection, log-phases, peaking parasitaemia, and later TPs indicated for blood and bone marrow sample collections. Necropsy endpoints (*) were planned for each animal well after day 14, when the natural decline in parasitaemia was expected, as observed for the three kra monkeys with chronic infections in E07 (Fig. S1), and when parasitaemia was still undetectable. Bottom Panel: Schematic showing E35 experimental data including P. knowlesi cryopreserved sporozoite inoculations on day 0 (syringe), daily parasitaemias graphed (pink lines), and defined TPs (gold bars) and the specific days of euthanasia and necropsy endpoints (*) are indicated for each of the animals. No treatment was required. [file 12936_2021_3925_MOESM12_ESM.png]

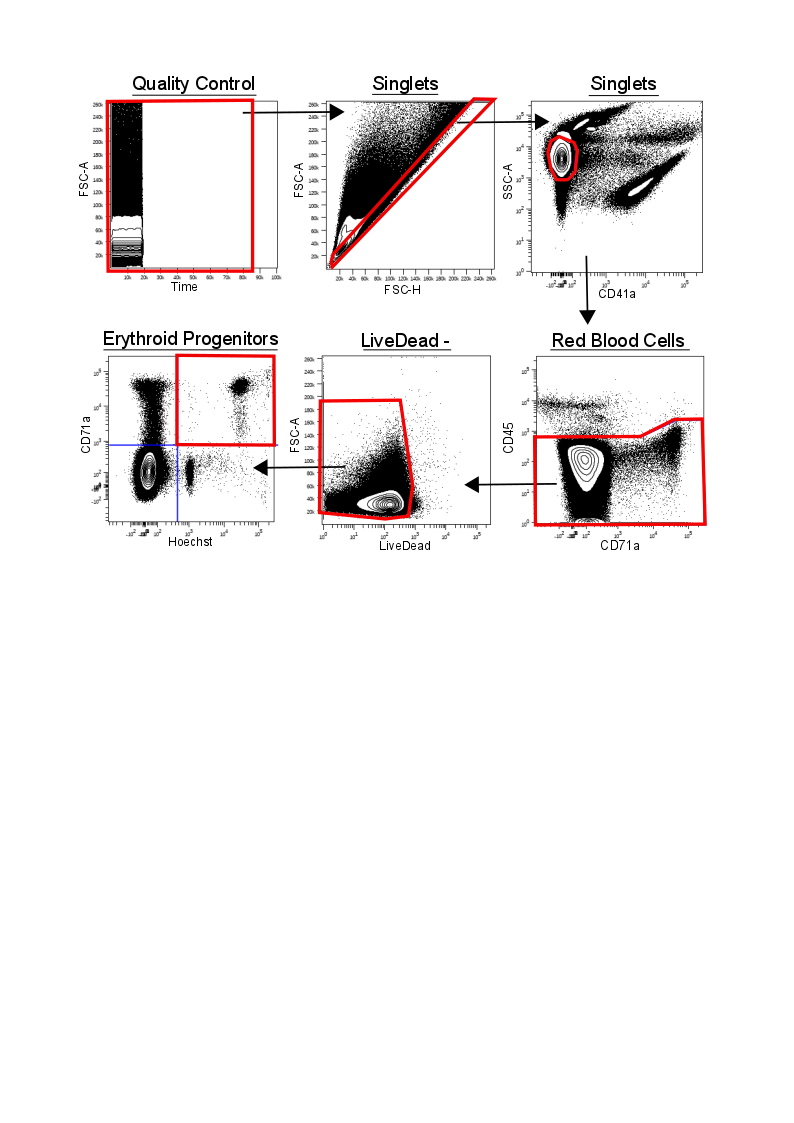

Supplement: Supplementary file 13 — Additional file 13: Fig. S4. Flow Cytometry Gating Strategy. Representative flow cytometry gating strategy for measuring erythroid lineage cells in bone marrow aspirate collected from the kra monkeys. [file 12936_2021_3925_MOESM13_ESM.png]

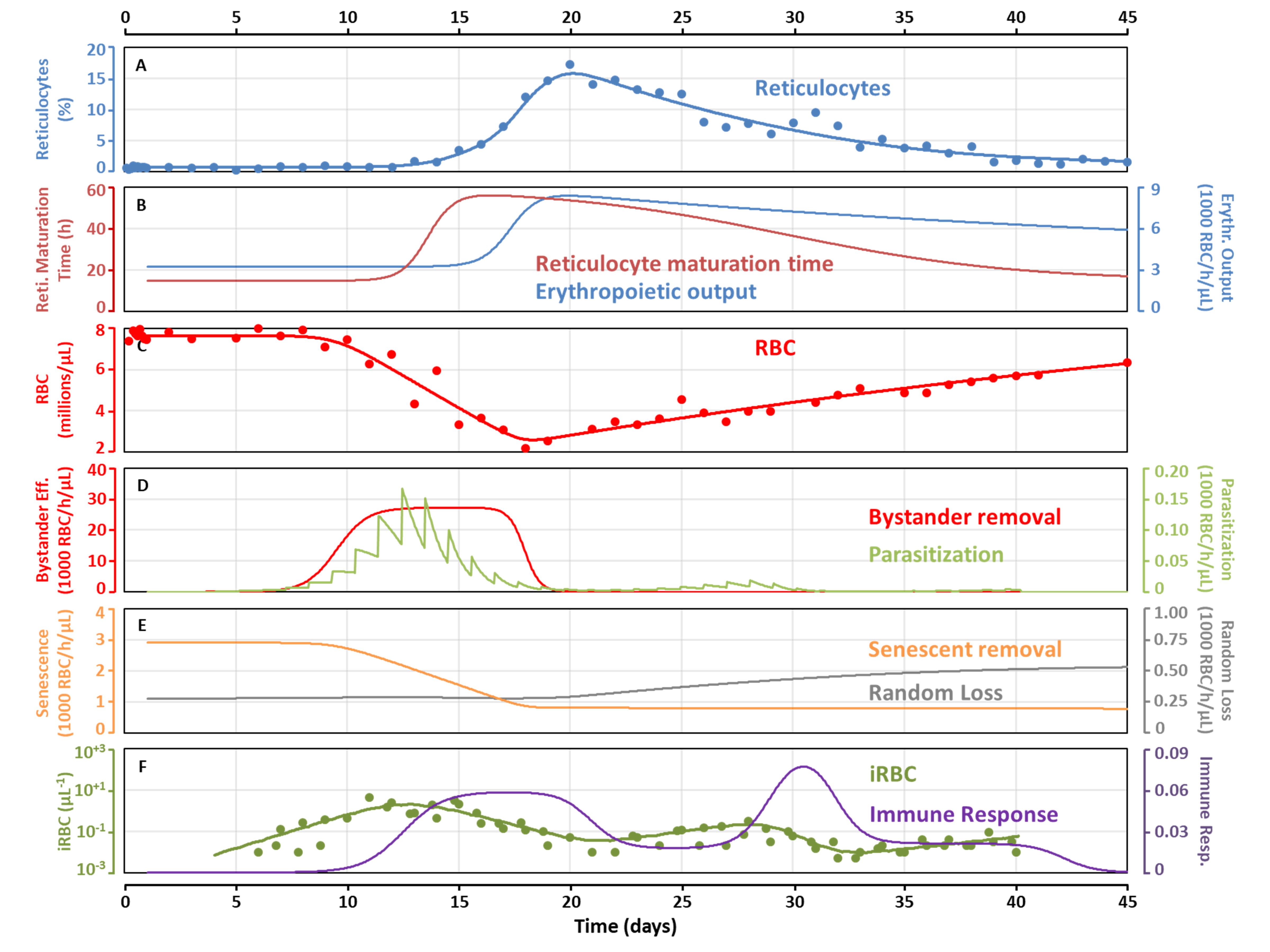

Supplement: Supplementary file 14 — Additional file 14: Fig. S5. Characterization of the dynamics of RBC removal and production processes in a representative kra monkey (12C44) during a P. knowlesi infection. To quantify the haemodynamic processes during a P. knowlesi infection, a computational dynamical model was used that was previously developed to faithfully track the blood dynamics in Plasmodium infected monkeys [63–65]. The model was formulated as a set of discrete recursive equations, where the pools of reticulocytes, RBCs, and iRBCs were stratified into age classes. The model directly represents that reticulocytes are released from the bone marrow with a certain age and rate, circulate for a day and then mature into RBCs. Pertinent model results (lines) are superimposed on experimental data (symbols). Shown are the circulating reticulocytes (A), mature RBCs (C), and infected RBCs (F), from which the model allowed the quantification of different causes of RBC removal (D and E). The NHP RBCs normally die after about 100 days due to senescence, or on a daily basis due to “random” effects, such as shear stresses (E). During Plasmodium infections, some of the healthy RBCs are also infected by merozoites and destroyed when the parasites are released (parasitisation, D) or lost to a bystander effect (D). Interestingly, large numbers of RBCs were lost during the infection due to the bystander mechanism (D). The profile of RBCs (C) demonstrates that the kra monkey had severe anaemia and responded appropriately by increasing the erythropoietic output and releasing younger reticulocytes, thereby increasing the reticulocyte maturation time in circulation (B). [file 12936_2021_3925_MOESM14_ESM.png]
